# Characterization of the First Cultured Psychrotolerant Representative of *Legionella* from Antarctica Reveals Its Unique Genome Structure

**DOI:** 10.1128/Spectrum.00424-21

**Published:** 2021-10-20

**Authors:** Sho Shimada, Ryosuke Nakai, Kotaro Aoki, Sakae Kudoh, Satoshi Imura, Norifumi Shimoeda, Giichiro Ohno, Kentaro Watanabe, Yasunari Miyazaki, Yoshikazu Ishii, Kazuhiro Tateda

**Affiliations:** a Department of Microbiology and Infectious Diseases, Toho University School of Medicine, Tokyo, Japan; b Bioproduction Research Institute, National Institute of Advanced Industrial Science and Technology (AIST), Hokkaido, Japan; c Department of Respiratory Medicine, Tokyo Medical and Dental University (TMDU), Tokyo, Japan; d National Institute of Polar Research, Research Organization of Information and Systems, Tokyo, Japan; e Department of Polar Science, The Graduate University for Advanced Studies, SOKENDAI, Tokyo, Japan; f Tochigi Medical Center, Tochinoki Hospital, Tochigi, Japan; g Tokatsu Hospital, Chiba, Japan; Nanyang Technological University

**Keywords:** *Legionella*, psychrotolerant, insertion sequence, horizontal gene transfer, comparative genomic analysis, cellular fatty acids, mobile genetic elements

## Abstract

Culture-independent analysis shows that *Legionella* spp. inhabit a wide range of low-temperature environments, but to date, no psychrotolerant or psychrophilic strains have been reported. Here, we characterized the first cultivated psychrotolerant representative, designated strain TUM19329^T^, isolated from an Antarctic lake using a polyphasic approach and comparative genomic analysis. A genome-wide phylogenetic tree indicated that this strain was phylogenetically separate at the species level. Strain TUM19329^T^ shared common physiological traits (e.g., Gram-negative, limited growth on buffered charcoal-yeast extract α-ketoglutarate [BCYEα] agar with l-cysteine requirements) with its relatives, but it also showed psychrotolerant growth properties (e.g., growth at 4°C to 25°C). Moreover, this strain altered its own cellular fatty acid composition to accumulate unsaturated fatty acid at a lower temperature, which may help maintain the cell membrane fluidity. Through comparative genomic analysis, we found that this strain possessed massive mobile genetic elements compared with other species, amounting to up to 17% of the total genes. The majority of the elements were the result of the spread of only a few insertion sequences (ISs), which were spread throughout the genome by a “copy-and-paste” mechanism. Furthermore, we found metabolic genes, such as fatty acid synthesis-related genes, acquired by horizontal gene transfer (HGT). The expansion of ISs and HGT events may play a major role in shaping the phenotype and physiology of this strain. On the basis of the features presented here, we propose a new species—Legionella antarctica sp. nov.—represented by strain TUM19329^T^ (= GTC 22699^T^ = NCTC 14581^T^).

**IMPORTANCE** This study characterized a unique cultivated representative of the genus *Legionella* isolated from an Antarctic lake. This psychrotolerant strain had some common properties of known *Legionella* species but also displayed other characteristics, such as plasticity in fatty acid composition and an enrichment of mobile genes in the genome. These remarkable properties, as well as other factors, may contribute to cold hardiness, and this first cultivated cold-tolerant strain of the genus *Legionella* may serve as a model bacterium for further studies. It is worth noting that environmentally derived 16S rRNA gene phylotypes closely related to the strain characterized here have been detected from diverse environments outside Antarctica, suggesting a wide distribution of psychrotolerant *Legionella* bacteria. Our culture- and genome-based findings may accelerate the ongoing studies of the behavior and pathogenicity of *Legionella* spp., which have been monitored for many years in the context of public health.

## INTRODUCTION

*Legionella* spp. are aerobic Gram-negative intracellular bacteria that mainly inhabit aquatic environments. Their natural hosts are protozoa such as free-living amoebae, while the accidental introduction of *Legionella* into humans via aerosols can cause pneumonia or fever-like symptoms known as Pontiac fever. More than 60 species of *Legionella* have been identified to date (1), around half of which are pathogenic to humans. Outbreaks of disease in human-made environments have been reported worldwide (2), and *Legionella* spp. are environmental bacteria that requires public health attention.

The *Legionella* species described to date are mesophilic, with an optimal growth temperature ranging from 25°C to 45°C (3, 4), none of which are psychrotolerant or psychrophilic. However, recent culture-independent analysis has elucidated the unexpected diversity of *Legionella* in low-temperature-treated drinking water, as well as polar lakes (5, 6). Our study also identified multiple uncharacterized *Legionella* lineages, sharing low 16S rRNA gene sequence similarities with known species in Antarctic terrestrial environments (7). From a public health perspective, the cultivation of uncultured *Legionella* species inhabiting cold environments is a crucial step in assessing the cold-tolerant mechanism(s) and potential pathogenicity of this organism.

In this context, we succeeded in isolating and cultivating the first psychrotolerant strain, designated TUM19329^T^, from Antarctic freshwater lake sediment (7). Here, our study physiologically and genomically characterized this newly discovered strain and compared its characteristics to other *Legionella* species. We consequently propose the underlying mechanism responsible for cold hardiness and describe a novel species—Legionella antarctica—for which this strain is representative.

## RESULTS AND DISCUSSION

### Phylogenetic position of strain TUM19329^T^ and related strains.

**FIG 1:**
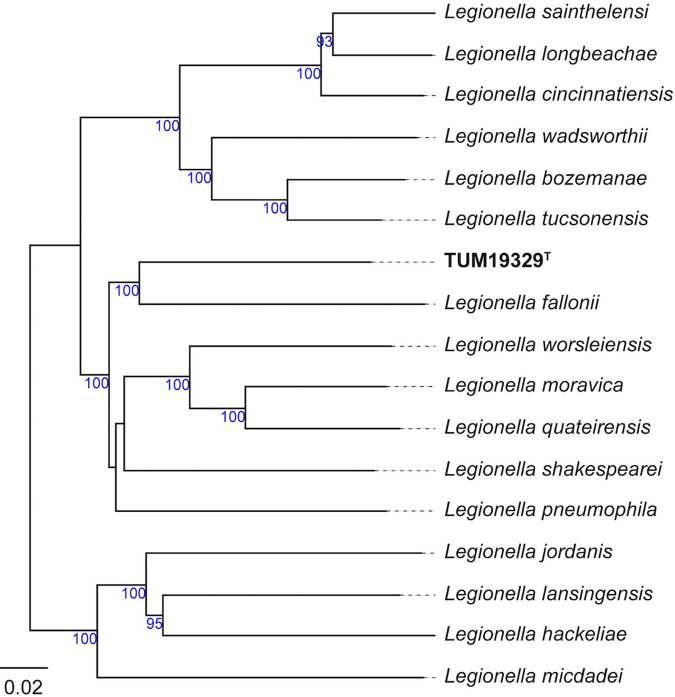
Tree inferred with FastME v2.1.6.1 from whole-proteome-based Genome BLAST Distance Phylogeny (GBDP) distances. The branch lengths are scaled via the GBDP distance formula *d*_5_. Branch values are GBDP pseudobootstrap support values of >60% from 100 replications, with an average branch support of 92.1%. The trees were rooted at the midpoint. The scale bar indicates the branch length value. The strain names of each species are listed in Table 2.

The three full-length 16S rRNA gene sequences (1,544 bp) of strain TUM19329^T^, which were retrieved from the genome (8), showed 97.4% to 97.5% sequence identities with the closest strain, Legionella fallonii LLAP-10^T^ (DDBJ/ENA/GenBank accession number LN614827; 9), in a BLASTN search against the NCBI nucleotide collection (nonredundant/nucleotide [nr/nt]) database as of July 2021. According to the identity threshold (<98.65%) for differentiating two species (10), this suggests that strain TUM19329^T^ is a candidate for a novel species. Note that the genome-wide average nucleotide identity (ANI) between strains TUM19329^T^ and LLAP-10^T^ was also relatively low at 77.5% (detailed comparative genomic comparisons are discussed later). Further phylogenetic analysis based on the genome sequences of strain TUM19329^T^ and other *Legionella* species also revealed that TUM19329^T^ was relatively close to *L. fallonii* on the phylogenetic tree (Fig. 1). The phylogenetic position in this tree is similar to the trend shown by the 16S rRNA gene sequence-based tree, which places strain TUM19329^T^ relatively close to *L. fallonii* (see Fig. S1 in the supplemental material), although this tree has potentially low resolution because of the short sequence information compared with the genome-scale tree (Fig. 1).

Strain TUM19329^T^ was distantly related to all known *Legionella* species, while a BLASTN database search against uncultured/environmental sequences showed that the sequences of this strain were almost identical (99.9% to 100%) to those recovered from a benthic moss colony known as “moss pillars” of another freshwater lake in Antarctica (GenBank accession number AB630760; 11). Moreover, on the basis of the IMNGS platform (12), which is the database search against metagenome-derived 16S rRNA gene amplicon data sets, we further found that the TUM19329^T^ 16S rRNA gene sequence matched 841 data sets with a sequence similarity threshold of 99%; the data sets contained 259 soil, 100 aquatic, 86 freshwater, 76 rhizosphere, and 76 Arabidopsis thaliana and other plant metagenomes. This data set includes sequence data derived from samples such as soil crust in the Svalbard (Arctic Norway) (accession number DRR148112), high-latitude Qinghai Lake sediments (SRR1303671), and lake water of Little Long Lake (SRR2962948; note that the temperature at the time of sample collection was around 20°C; 13). The habitats of this strain inferred using the ProkAtlas tool (containing multiple 16S rRNA gene sequences labeled by one environmental category [14] with a threshold of 99%) were permafrost (habitat preference score, 25.0%), rice paddy (21.5%), freshwater (19.2%), soil (18.8%), and wetland (15.4%). The results suggested that the relatives of this strain may be distributed not only in Antarctic aquatic habitats but also in a variety of other environments.

### Morphological, physiological, infectious, and biochemical characteristics.

TUM19329^T^ is a Gram-stain-negative, non-spore-forming bacterium with a size of 2.2 by 0.3 μm. Growth was observed at 4°C to 25°C but not above 30°C on buffered charcoal-yeast extract α-ketoglutarate (BCYEα) agar. Colonies appeared after 10 days at 25°C. Given such cold-tolerant growth characteristics, initial enrichment cultivation at 4°C rather than 33°C, which is the general growth condition set for previously described *Legionella* spp., may have led to successful isolation of the first psychrotolerant strain. Note that the temperature of the freshwater lake, known as Lake Naga-ike, which strain TUM19329^T^ inhabits, ranges from 0°C to 10°C (15). The strain only grew at pH 6.5 to 7.0, with a NaCl concentration lower than 1%, in buffered yeast extract (BYE) broth (Fig. S2). The strain required l-cysteine for growth. Both cefinase and oxidase tests were negative, as were gelatinase, hippurate reactions, and all the sugar tests.

**FIG 2:**
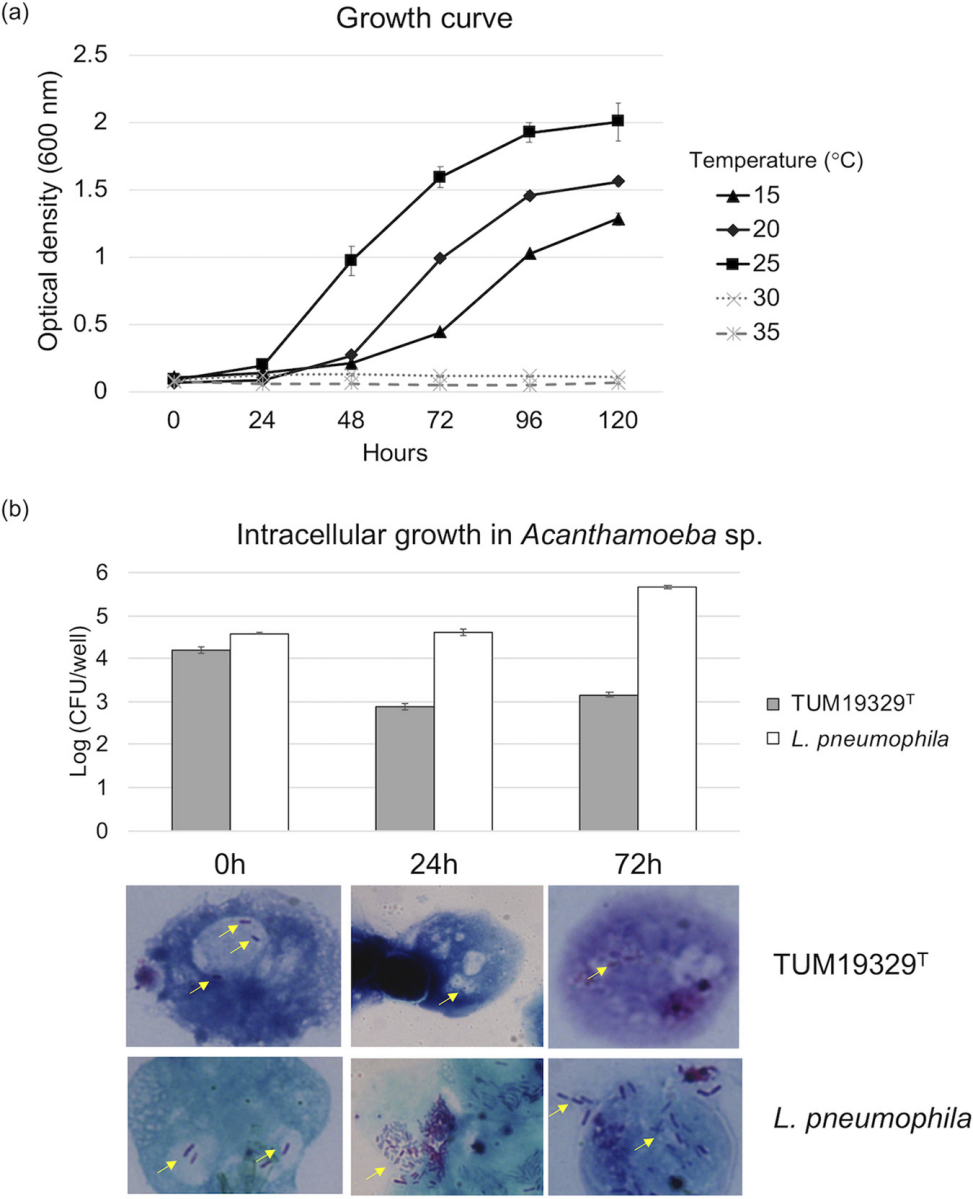
Proliferative potential of strain TUM19329^T^ in culture medium and eukaryotic cells. (a) Growth curves of strain TUM19329^T^ in BYE broth at different temperatures. (b) Intracellular growth of strain TUM19329^T^ and L. pneumophila Philadlphia-1 in an *Acanthamoeba* sp. The light micrograph images were obtained by Gimenez staining at each time point. All images were taken at ×1,000 magnification. The yellow arrows indicate bacterial cells. The experiment was conducted twice in triplicate.

To assess the replication capacity of TUM19329^T^ in host eukaryotes, we compared its intracellular growth with that of Legionella pneumophila strain Philadelphia-1 in *Acanthamoeba* spp., a known natural host of *Legionella* spp. Whereas L. pneumophila tended to proliferate after intracellular uptake, the bacterial number of strain TUM19329^T^ decreased after 24 h of uptake, and thereafter, no change was observed until 72 h (Fig. 2b). Although our strain did not show such clear proliferation, there may be a different and more suitable host for this strain in cold environments. We propose that our psychrotolerant strain may be used as bait to screen for and identify its potential host(s). Pursuing its host will accelerate our understanding of the ecology of *Legionella* spp. under low-temperature conditions.

### Chemotaxonomic characteristics.

The G+C content of the genome of strain TUM19329^T^ was 39.1%, and the major respiratory quinones were Q-12 (69.1%) and Q-13 (30.9%); these features were shared with the closest strain, *L. fallonii* LLAP-10^T^. The dominant cellular fatty acids (CFAs; >10% of the total fatty acids) of the strain grown at 25°C were anteiso-C_15:0_ (36.2%), C_16:1_*ω*7*c*/C_16:1_*ω*6*c* (27.2%), and iso-C_16:0_ (15.2%) (Table 1). The pattern of large amounts of branched CFAs was also similar to that observed in *L. fallonii* LLAP-10^T^ and its close relatives (9); however, the CFA profile drastically changed when cultured at lower temperature. After cultivation at 10°C, the proportion of monounsaturated fatty acids (C_16:1_*ω*7*c*/C_16:1_*ω*6*c*) among the total fatty acids changed from 27.2% to 53.7% (Table 1).

**TABLE 1:** CFA compositions of strain TUM19329^T^ and the most closely related strain, LLAP-10^T^^*a*^

Fatty acid or characteristic	Data for strain:		
	TUM19329^T^		LLAP-10^T^
Cultured temp (°C)	10	25	30
iso-C_13:0_	–	T	–
anteiso-C_13:0_	–	T	–
iso-C_14:0_	1.3	3.6	4-5
C_14:1_*ω*5*c*	1.1	1.2	–
C_14:0_	1.1	T	2-3
C_13:0_ 3-OH / C_15:1_ iso H (summed feature 1)	–	T	ND
iso-C_15:0_	1.5	3.9	T
anteiso-C_15:0_	19.9	36.2	12–15
C_15:1_ω6c	1.6	1.6	3–4
C_15:0_	–	–	2–3
C_14:0_ 3-OH	T	T	–
iso-C_16:0_	9.0	15.2	11–13
C_16:1_*ω*7*c*/C_16:1_*ω*6*c* (summed feature 3)	53.7	27.2	21–27^*b*^
C_16:0_	4.2	1.7	15–19
C_15:0_ 2-OH	–	T	–
iso-C_17:1_*ω*9*c*/C_16:0_ 10-methyl (summed feature 9)	T	T	ND
iso-C_17:0_	T	T	–
anteiso-C_17:0_	3.0	4.1	2–3
C_17:0_ cyc	–	–	2–6
C_17:0_	–	T	2
iso-C_18:0_	–	T	T
C_18:0_	1.8	1.0	6-7
C_18:0_ 3-OH	–	T	ND
C_19:0_ cyc *ω*8*c*	–	T	ND
C_19:0_	–	–	1–2
anteiso-C_19:0_	–	–	T
C_20:0_	T	T	1–2

a Values are the percentage of total fatty acids. T, trace (∼0.9%); –, not detected; ND, no data. Data of strain LLAP-10^T^ from Adeleke et al. (9). Note that the psychrotolerant Antarctic strain TUM19329^T^ from this study does not grow above 30°C.

b This is given as the value of C_16:1_ *ω*7*c* in the previous study.

One of the prominent biological strategies to survive at low temperature is believed to be the maintenance of cell membrane fluidity (16). Generally, a decrease in temperature causes membrane viscosity or phase transition in biological membranes due to the tight packing of the fatty acyl chains (17). To overcome this, cold-adapted organisms alter fatty acid production and contain unsaturated fatty acyl chains containing one or more double bonds, which adopt a more expanded conformation and possess a lower melting temperature than their corresponding saturated chains (17); for example, a deep-sea psychrotolerant bacterium Shewanella electrodiphila MAR441^T^ accumulates monounsaturated fatty acids (e.g., C_16:1_*ω*7*c* and C_18:1_*ω*7*c*) to more than half (56%) of the total fatty acids at low temperature (18). Taken together, we believe that the ability of strain TUM19329^T^ to alter its own fatty acid content is one of the crucial mechanisms of cold tolerance.

### Comparative genome analysis.

**General overview.** We further performed a comparative genomic analysis with 16 known mesophilic *Legionella* species to characterize the genomic properties of the cold-tolerant strain TUM19329^T^. The general features of strain TUM19329^T^ compared to other species are summarized in Table 2. Previous comparative genomic studies have shown that some psychrophilic and psychrotolerant bacteria tend to have distinctive genomic features (e.g., altered G+C contents) and amino acid usage frequencies (e.g., reduced proline contents) (19–22). However, in our comparative genomic analysis, although the *Legionella* species compared showed some differences in genome size (ranging from 2.99 to 4.43 Mb), G+C content (36.9 to 41.7%), and protein-coding sequence (CDS) number (2,703 to 3,761), strain TUM19329^T^ did not have the distinct characteristics reported in previous studies (see details below).

**TABLE 2:** Summary of the basic genomic data of the 17 *Legionella* spp. compared in this study

Taxon name	Strain name	GenBank accession ID	No. of contigs	Size (Mbp)	GC (%)	No. of CDS	No. of rRNA	No. of tRNA
*Legionella antarctica*	TUM19329^T^	GCA_011764505.1	1	3.75	39.15	3,608	9	42
Legionella bozemanae	WIGAT	GCA_900450575.1	7	4.23	37.97	3,761	9	46
Legionella cincinnatiensis	72-OH-H^T^	GCA_900452415.1	2	4.1	36.86	3,498	9	46
Legionella fallonii	LLAP-10^T^	GCA_000953135.1	3	4.43	38.32	3,748	12	46
Legionella hackeliae	Lansing 2^T^	GCA_000953655.1	2	3.57	38.96	3,183	12	43
Legionella jordanis	BL 540^T^	GCA_900637635.1	1	3.13	41.74	2,865	12	44
Legionella lansingensis	1677-MI-H^T^	GCA_900187355.1	1	2.99	40.7	2,703	12	43
Legionella longbeachae	Longbeach 4^T^	GCA_004283175.1	38	4.13	37	3,554	5	42
Legionella micdadei	TATLOCK^T^	GCA_000953635.1	1	3.31	40.46	2,953	9	43
Legionella moravica	316-36^T^	GCA_900452715.1	2	3.83	40.12	3,212	9	41
Legionella pneumophila subsp. *pneumophila*	Philadelphia-1	GCA_001752765.1	1	3.41	38.33	3,021	9	43
Legionella quateirensis	200/83-1335^T^	GCA_900452695.1	5	4.28	39.06	3,611	9	43
Legionella sainthelensi	MSH-4^T^	GCA_900637685.1	1	4.16	37.06	3,746	12	49
Legionella shakespearei	214^T^	GCA_001468025.1	76	3.51	41.56	2,933	4	40
Legionella tucsonensis	1087-AZ-H^T^	GCA_001468035.1	12	3.36	37.41	2,967	3	41
Legionella wadsworthii	Wadsworth 81-716A^T^	GCA_900452925.1	2	3.6	38.08	3,189	11	43
Legionella worsleiensis	95/83-1347^T^	GCA_900453045.1	2	3.16	40.4	2,705	9	41

**FIG 3:**
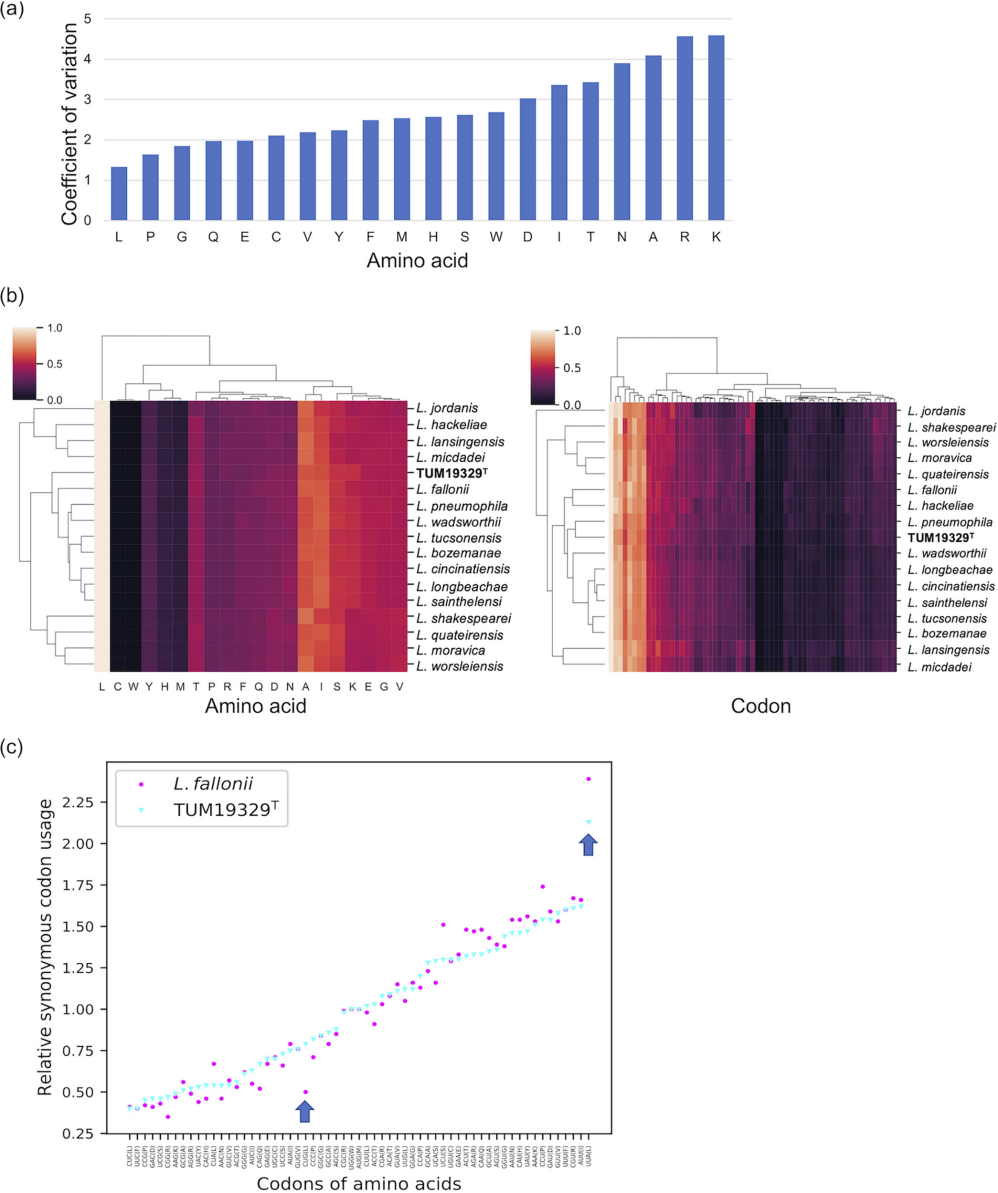
Comparison of the amino acid and codon composition of 17 *Legionella* species. (a) Coefficient of variation of the amino acid compositions between the compared *Legionella* species. (b) An amino acid and codon usage heatmap based on the percentages in each of the genomes of the compared *Legionella* species. A heatmap describing the details of each codon on the horizontal axis is available in Figshare (see “Data Availability”). The strain names of each species are listed in Table 2. (c) Relative synonymous codon usage (RSCU) in TUM19329^T^ and *L. fallonii*. Stop codons were excluded from the analysis. Codons of amino acids in the *x* axis were sorted based on the RSCU values of TUM19329^T^. The amino acids translated by codon are shown in parentheses following the codon. The arrows point to the synonymous codon for leucine, which showed the most usage bias between the two species.

Specifically, the codon usage and amino acid composition of the 17 species were relatively similar (Fig. 3a), and the strains that were related to each other tended to form close clusters on the hierarchically clustered heatmap based on the percentage of codons and amino acids of the entire genome (Fig. 3b). TUM19329^T^ was placed close to its phylogenetic relative, *L. fallonii*, in the heatmap based on amino acid usage (Fig. 3b, left panel). Meanwhile, in terms of codon usage, TUM19329^T^ was close to L. pneumophila (Fig. 3b, right panel), thus differing from the trend observed in the comparison of amino acid composition. This suggested that there is a difference in the frequency of codon usage between TUM19329^T^ and *L. fallonii*. Comparing the two species for relative synonymous codon usage, TUM19329^T^ tended to use CUG more often, whereas *L. fallonii* tended to use UUA to encode leucine (Fig. 3c). To understand whether this genus changes its codon/amino acid usage as part of a cold adaptation strategy, a further collection of cold-tolerant *Legionella* strains is needed.

As one of the other possible factors for cold hardiness, cold shock proteins are generally essential single-stranded nucleic acid binding proteins that respond to cold shock and regulate a variety of cellular processes (23). The TUM19329^T^ genome possessed four cold shock proteins encoded by the genes TUM19329_00491, TUM19329_01544, TUM19329_02137, and TUM19329_03456. However, the other 16 mesophilic species also possessed four to six cold shock protein-coding genes. Furthermore, three out of the four cold shock proteins of TUM19329^T^ formed part of the core genome of the 17 species, and the remaining gene was also shared between several species, suggesting that these proteins are well preserved among *Legionella* spp. In conclusion, TUM19329^T^ showed no specificity in the number or sequence of cold shock proteins compared with the mesophilic species. These results suggest that factors different from the well-known genomic characteristics and cold shock proteins of psychrophilic/psychrotolerant microorganisms contribute to its cold-tolerant nature. We further compared the individual genes among species to search for features that may explain the psychrotolerant characteristics of this strain.

### Core genome.

The pan-genome of 17 *Legionella* species consisted of 11,135 pan-genome orthologous groups and 1,340 core orthologous groups. The core orthologous groups consisted of the genes of fundamental metabolic pathways, as well as the Dot/Icm-type IVB secretion system and the type II secretion system, which are essential for intracellular replication. Like other *Legionella* species, these genes were conserved in TUM19329^T^, although duplicate genes were observed for *icmD*, similar to its most closely related species, *L. fallonii* (24). Interestingly, the region in which the *icmR* gene is located in L. pneumophila was replaced with genes that were nonhomologous to the *icmR* gene (TUM19329_00496) in TUM19329^T^. IcmR, the gene for which is located between *icmS* and *icmQ*, interacts with IcmQ as a chaperone to block the dimeric form of IcmQ (25). IcmQ is well conserved among *Legionella*, whereas IcmR is hypervariable depending on the species and is thought to be encoded by a fast-evolving gene (26). However, these genes are referred to as functional homologs of IcmRs (FIRs) because they perform the same functions as IcmR. A previous study showed that phylogenetically closely related species mostly share homologous FIRs (27). Indeed, phylogenetic analysis of the FIR gene sequences showed that several phylogenetically related strains (see Fig. 1) formed groups supported with high bootstrap values (∼70%; e.g., the group of L. tucsonensis, L. wadsworthii, and L. bozemanae; Fig. S3). In contrast, the putative FIR gene of strain TUM19329^T^ had the closest phylogenetic relationship with that of *L. fallonii* among the known species, but its bootstrap value was low. Furthermore, direct comparison of the sequences of strain TUM19329^T^ and *L. fallonii* by BLASTP showed low similarity and coverage of 37% and 41%, respectively. Consistent with the findings observed for other *Legionella* spp., the FIR of TUM19329^T^ may also have changed in the process of species diversification.

### Massive insertion sequences (ISs) and putative horizontal gene transfer (HGT).

**FIG 4:**
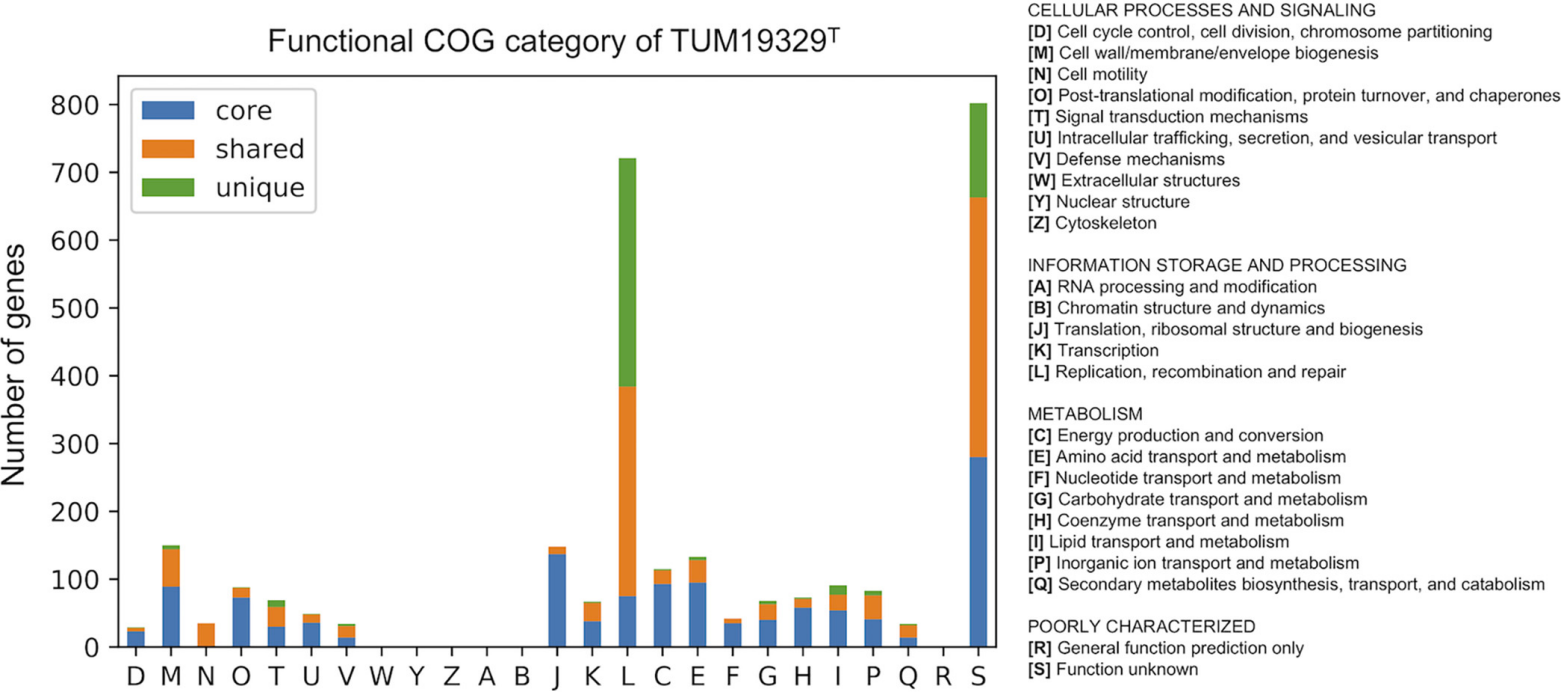
Number of protein coding genes classified into each functional COG category. Genes belonging to each category are colored as core, shared (genes shared between at least one species), or unique. Protein coding genes not annotated with any functional category (729 CDS, 20%) or annotated with multiple COG functional categories (46 CDS, 1.2%) were excluded. COG, clusters of orthologous groups.

**FIG 5:**
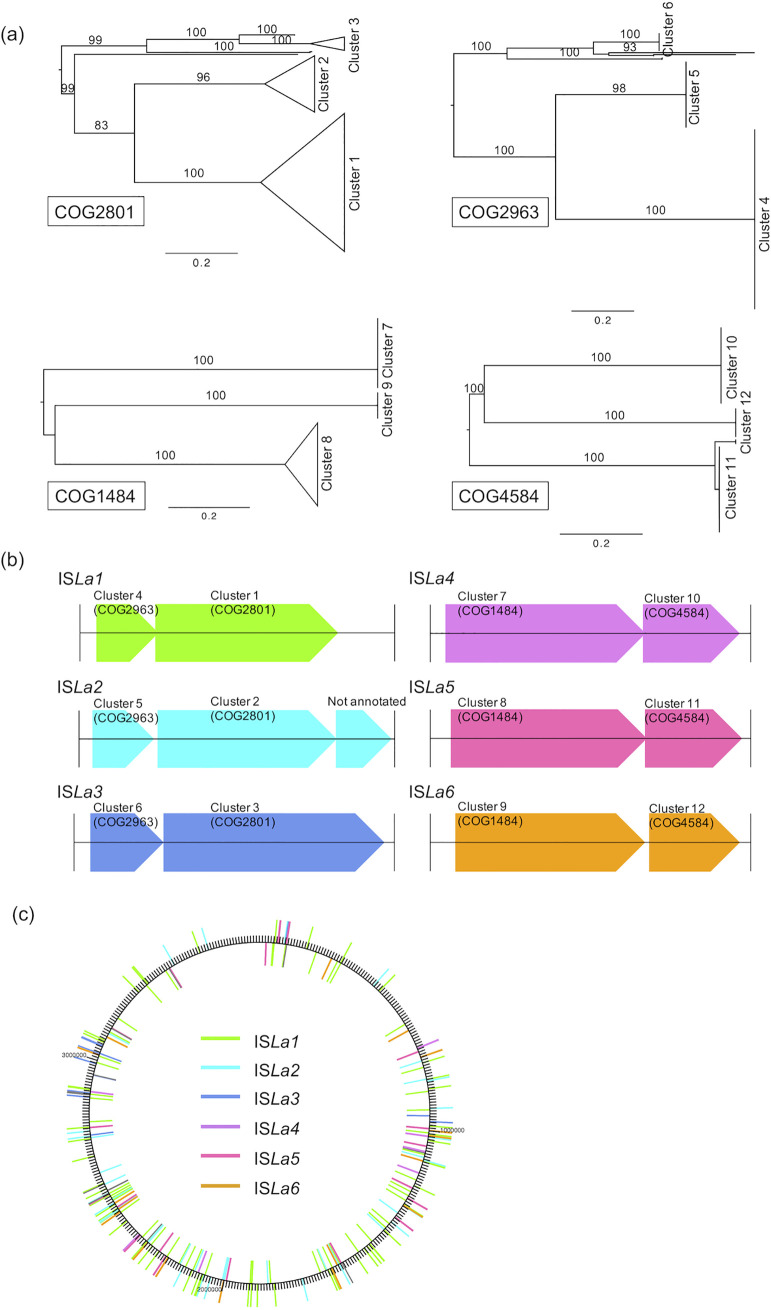
Phylogeny, structure, and distribution of the insertion sequences in the genome of TUM19329^T^. (a) Maximum-likelihood tree showing the relationship between the genes classified into the four COG IDs with the highest number of genes, COG2801, COG2963, COG1484, and COG4584. The tree was rooted using midpoint rooting. The scale bar represents the branch length value. Numbers along branches are bootstrap values based on 100 replicates. The genes in each COG ID were grouped in three major clusters. Detailed versions of each tree are available in Figshare (see “Data Availability”). (b) Each of the 12 clusters appeared adjacent to each other in a specific pair in the genome, and analysis, which included the up- and downstream sequences, revealed six IS structures (IS*La1* to IS*La6*) that appeared repeatedly. (c) The distribution of IS*La1* to IS*La6* in the genome of TUM19329^T^.

Regarding the number of assigned functional genes, 2,879 CDSs of TUM19329^T^ (79.7%) were annotated to the cluster of the orthologous group (COG) category. While most of the genes in each functional category were core or shared genes among the 17 species, TUM19329^T^ had a significantly higher proportion of genes classified to the replication, recombination, and repair [L] category, which accounted for 20% (721 genes) of the entire genome (Fig. 4, Table S1) compared with other *Legionella* species (*n* = 120 to 220, 3.8% to 5.9%). The composition of the COGs classified as [L] was mostly mobile genetic elements (*n* = 614) such as transposases, integrases, and plasmids. Among these, the most commonly observed COG IDs were COG2801 (*n* = 163), COG2963 (*n* = 156), COG4584 (*n* = 45), and COG1484 (*n* = 44) (Table S2). To clarify the sequence relationships of the genes classified into these four COG IDs, they were further analyzed using the phylogenetic tree method. As a result, the gene sequences of each COG ID were clustered into a small number of groups (Fig. 5a). Looking carefully at the position of these sequences in the genome, we found that the genes in certain clusters always appeared in pairs (e.g., cluster 1 and cluster 4 in Fig. 5). As a result of the alignment of these genes, including the up- and downstream sequences, we found six copies (IS*La1* to IS*La6*) of the IS structure that encoded several genes and incomplete terminal inverted repeat structures at the end (Fig. 5b, Table 3). These ISs appeared repeatedly, and a total of 169 ISs containing a full-length copy were identified, distributed in various positions throughout the chromosome (Fig. 5c).

**TABLE 3:** Descriptions of the six IS structures with particularly high copy numbers

IS (length [bp])	No. of full-length copies	Gene^*a*^ (length [bp])	Description of the gene (COG ID)	BLAST similarity search^*b*^			Putative HGT^*c*^
				Host of the gene	Query coverage (%)	Identity (%)	
IS*La1* (1,463)	93	Cluster 4 (285)	Hypothetical protein (COG2963)	*Legionella* sp.	96	78.02	No
				Legionella geestiana	96	72.53	
				*Legionellaceae* sp.	97	70.65	
		Cluster 1 (846)	Transposase InsF for insertion sequence IS*3A* (COG2801)	*Legionella* sp.	100	98.58	Yes
				*Legionellaceae* sp.	98	82.31	
				Legionella pneumophila	98	82.31	
IS*La2* (1,480)	33	Cluster 5 (285)	Hypothetical protein (COG2963)	*Gammaproteobacteria* sp.	100	77.66	Yes
				*Desulfobacteraceae* sp. strain 4484_190.2	98	59.14	
				Desulfacinum hydrothermale	94	55.06	
		Cluster 2 (837)	Transposase for insertion sequence element IS*3411* (COG2801)	*Desulfobacula* sp. strain RIFOXYA12_FULL_46_16	99	65.34	Yes
				Desulfacinum infernum DSM 9756	97	66.05	
				*Nitrospirae* sp.	97	65.56	
		(258)	Hypothetical protein (not annotated)	“*Candidatus* Kentron” sp. TC	58	49.06	No
				“*Candidatus* Kentron” sp. TC	31	70.37	
				“*Candidatus* Kentron” sp. LFY	28	70.83	
IS*La3* (1,260)	6	Cluster 6 (291)	Transposase InsN for insertion sequence element IS*911A* (COG2963)	*Legionella* sp.	100	98.58	Yes
				*Legionellaceae* sp.	98	82.31	
				Legionella pneumophila	98	82.31	
		Cluster 3 (876)	Insertion element IS*600* uncharacterized 31 kDa protein (COG2801)	*Legionella* sp.	97	95.09	Yes
				Legionella pneumophila strain Leg01/53	100	73.29	
				Legionella pneumophila	100	73.04	
IS*La4* (2,431)	17	Cluster 7 (1,512)	Transposase (COG1484)	Solemya velum gill symbiont	97	61.05	Yes
				Legionella taurinensis	100	56.26	
				Legionella taurinensis	100	56.26	
		Cluster 10 (729)	IS*232* putative ATP-binding protein (COG4584)	*Solemya velum* gill symbiont	100	65.29	Yes
				Legionella taurinensis	100	61.57	
				*Alphaproteobacteria* sp.	99	58.51	
IS*La5* (2516)	13	Cluster 8 (1,533)	Putative transposase for insertion sequence element IS*5376* (COG1484)	*Methylotenera* sp.	100	98.24	No
				*Legionella* sp.	99	73.87	
				*Sphingosinicella* sp.	99	73.67	
		Cluster 11 (756)	Putative ATP-binding protein in insertion sequence (COG4584)	*Methylotenera* sp.	100	98.8	Yes
				*Legionella* sp.	100	82.47	
				“*Candidatus* Saccharibacteria” sp.	97	77.96	
IS*La6* (2,631)	7	Cluster 9 (1,551)	Putative transposase for insertion sequence IS*408* (COG1484)	*Gammaproteobacteria* sp.	100	74.08	Yes
				*Coxiellaceae* sp.	98	53.24	
				Deltaproteobacteria sp.	98	51.95	
		Cluster 12 (738)	Insertion sequence IS*408* putative ATP-binding protein (COG4584)	*Gammaproteobacteria* sp.	100	82.86	Yes
				*Aquisalimonas* sp. strain 2447	97	56.25	
				“*Candidatus* Kentron” sp. DK	94	57.94	

^*a*^Cluster number defined based on the results of phylogenetic analysis of the same COG ID (see Fig. 5a).

^*b*^The results of the top three hits of the BLASTP similarity search for each gene.

^*c*^Genes presumed to have been acquired by HGT using HGTector.

ISs are the major driver of genome evolution by interrupting gene functions and reshaping the genome structure by recombination (28). We found multiple IS structures that were fragmented by other ISs (Table S3, Fig. S4). We also identified some of the functional genes other than mobile genes flanking the ISs that were annotated with the same COG ID. These genes are also likely to be remnants of genes fragmented by ISs (Table 4). Considering that the ISs may also regulate the expression of other genes by modifying their promoter regions, the massive ISs found in TUM19329^T^ may play a major role in shaping the phenotype and physiology of this strain by interference with many of its genes.

**TABLE 4:** Genes interrupted by IS*La1* to IS*La6*

Genes^*a*^	COG ID	Product	IS interrupting the gene
TUM19329_00019, TUM19329_00022	L:COG0507	Hypothetical protein	IS*La5*
TUM19329_00269, TUM19329_00272	V:COG0286	Site-specific DNA-methyltransferase (adenine-specific)	IS*La4*
TUM19329_00485, TUM19329_00488	G:ENOG410XNQK	Arabinose-proton symporter	IS*La1*
TUM19329_00643, TUM19329_00646	S:COG0121	Putative glutamine amidotransferase	IS*La4*
TUM19329_00913, TUM19329_00917	I:COG3243	Poly(3-hydroxyalkanoate) polymerase	IS*La2*
TUM19329_00959, TUM19329_00962	I:COG1960	Acyl-CoA dehydrogenase family member 11	IS*La3*
TUM19329_01188, TUM19329_01191	V:COG1680	Serine-type d-Ala-d-Ala carboxypeptidase	IS*La1*
TUM19329_01427, TUM19329_01430	T:ENOG410XNMH	Histidine kinase	IS*La1*
TUM19329_01576, TUM19329_01580, TUM19329_01584	T:ENOG410XNMH	Histidine kinase	IS*La2*
TUM19329_01604, TUM19329_01607	S:ENOG4112B5D	Hypothetical protein	IS*La1*
TUM19329_01615, TUM19329_01618	S:ENOG4112A90	Protein TraM	IS*La1*
TUM19329_01623, TUM19329_01626	L:COG2189	Site-specific DNA-methyltransferase (adenine-specific)	IS*La1*
TUM19329_01804, TUM19329_01807	I:COG1835	*O*-acetyltransferase OatA	IS*La1*
TUM19329_02422, TUM19329_02428	M:COG2230	Cyclopropane-fatty-acyl-phospholipid synthase	IS*La2*
TUM19329_02454, TUM19329_02458	S:COG1988	Hypothetical protein	IS*La1*
TUM19329_02485, TUM19329_02491	M:COG0438	1,4-alpha-glucan branching enzyme	IS*La1*
TUM19329_02509, TUM19329_02512	S:COG3173	Hypothetical protein	IS*La1*
TUM19329_02618, TUM19329_02621	E:COG0028	Acetolactate synthase	IS*La1*
TUM19329_03112, TUM19329_03115	S:ENOG410XUV4	Protein BcsG-like protein	IS*La1*
TUM19329_03264, TUM19329_03267	I:COG1597	Undecaprenyl-diphosphate phosphatase	IS*La1*

a Mobile genes interrupted by ISs are excluded from the list.

Expansion of ISs, as seen in this strain, has been reported in other bacteria and is recognized to play an important role in niche-restricted prokaryotes, such as extremophiles and symbionts, aiding their adaptation to the environment (28). For extremophiles, the enrichment of transposase-encoding genes has been reported in species residing in cold environments, such as Methanococcoides burtonii isolated in Antarctica (29). Furthermore, transposases were one of the overrepresented genes detected in metagenomic analysis of cold, 4,000-m-deep ocean samples (30). For symbionts, the expansion of ISs has been observed, especially in species that recently adopted a host-restricted lifestyle compared with long-standing symbionts (31, 32). From an evolutionary viewpoint, this phenomenon has been proposed as a hypothesis that the expansion of ISs reorganizes the genome in the early stages of the evolutionary adaptation process and that unnecessary genes are gradually eliminated, thereby streamlining the genome. In accordance with this hypothesis, strain TUM19329^T^ might be undergoing genome reduction and streamlining while adapting to its host in a cold environment.

For the detected genes in ISs, a similarity search using BLASTP with default settings (accessed July 2021) confirmed that 9 of the 13 genes were not carried by other *Legionella* spp. (Table 3). Furthermore, the other four genes that had best hits with *Legionella* spp. were found to be acquired by HGT based on a search using HGTector 2 (33), an automated pipeline determining putative horizontally transferred genes. HGTector 2 detects HGT by analyzing BLAST hit distribution patterns based on defined hierarchical evolutionary categories (see Materials and Methods). In accordance with the BLASTP similarity and the results of HGTector 2, 12 out of the 13 genes were likely to have been acquired by HGT relatively recently, rather than being derived from a common ancestor of the genus *Legionella*.

We further searched for genes that this strain may have acquired by HGT events across the whole genome. The number of detected putative genes acquired by HGT was 605 in total, which was higher than for other *Legionella* species (153 to 394 genes), but many of these were mobile genes (373 genes), such as transposases that form massive ISs. This further confirms that many of the mobile genes in this strain have been horizontally acquired and spread throughout the genome by the “copy-and-paste” mechanism.

### Unique fatty acid-related genes in a psychrotolerant Antarctic strain.

The majority of the high number of genes potentially acquired by HGT were mobile genes, but several putative HGT-derived genes related to metabolic pathways and functions were also identified. These genes were mainly classified into the following COG categories: cell wall/membrane/envelope biogenesis [M] (*n* = 25), amino acid transport and metabolism [E] (*n* = 16), energy production and conversion [C] (*n* = 14), inorganic ion transport and metabolism [P] (*n* = 12), lipid transport and metabolism [I] (*n* = 11), and transcription [K] (*n* = 10). It is possible that at least some of these putative exogenous genes were necessary for this strain to adapt to the low temperature conditions of the Antarctic lake.

In particular, several fatty acid synthesis-related genes in COG category [I] were overrepresented in this strain. In addition to the genes shared with other species, TUM19329^T^ possessed extra genes acquired by HGT annotated as *fabH* (TUM19329_01025), *fabG* (TUM19329_01799 and TUM19329_02687), *fabF* (TUM19329_03119), and *fabZ* (TUM19329_03121). These genes were presumed to be derived from members of the *Actinobacteria*, *Proteobacteria*, and other phyla. Moreover, an extensive BLASTP search against the NCBI nr database (accessed July 2021; option exclude “uncultured/environmental sample sequences”) revealed that, at least for *fabF* and *fabZ*, no *Legionella*-derived sequences were found in the top 100 sequences with high similarities. For example, the top-hit alignments of *fabF* and *fabZ* sequences were with those derived from “*Candidatus* Rubidus massiliensis” of the phylum *Chlamydiae* (54.0% sequence similarity) and Kibdelosporangium banguiense of the phylum *Actinobacteria* (41.1%), respectively. Judging from the results based on the HGTector and BLASTP searches, it is more likely that these two genes were exogenously acquired.

Interestingly, it has been proposed that the fatty acid metabolism of Coxiella burnetii, which is the etiologic agent of Q fever and belongs to the family *Coxiellaceae* of the order *Legionellales*, is enhanced by HGT-derived genes (34). Strain TUM19329^T^ also retains Fab-encoding gene sequences common to other *Legionella* species, and the retention of both conventional and potentially exogenous genes might be related to the enhancement of cold tolerance through fatty acid synthesis and alterations, although further verification is needed. Although the mechanisms remain to be resolved, this psychrotolerant strain is capable of the flexibility afforded by altering its CFA composition (Table 1), and future studies should examine the relationship between the expression of lipid synthesis-associated genes and the physiology of this strain under different culture conditions.

### Conclusion.

Unexplored *Legionella* members have been reported to exhibit high diversity in low-temperature habitats, but it remained unclear whether such strains possess true cold tolerance or whether they reside within a milder environment within their host protist(s). We characterized the first psychrotolerant strain, TUM19329^T^, from the sediment of an Antarctic lake. Our polyphasic approach shed light on the mechanism and potential factors responsible for the cold tolerance of this strain. On the basis of its physiological, phylogenetic, and chemotaxonomic properties, we propose naming this strain *Legionella antarctica* sp. nov.

### Description of *Legionella antarctica* sp. nov.

*Legionella antarctica* (ant.arc’ti.ca. L. fem. adj. *antarctica* southern, pertaining to the Antarctic, where the type strain was isolated).

Cells are 2.2 by 0.3 μm in size, Gram-negative, catalase-negative, oxidase-negative rods that require l-cysteine for growth on BCYEα. Smooth, gray colonies form after 7 to 10 days on BCYEα agar at 25°C, which show slight yellow fluorescence under UV light. Growth is observed at 4°C to 25°C (optimum, 25°C), but not at 30°C, and pH 6.5 to 7.0. No growth occurs in the presence of >1% NaCl. After 14 days of culture, cells test positive for acetoin production with the API 20E test and test positive for esterase, l-arginine arylamidase, and l-aspartic acid arylamidase with the API Campy test. The major fatty acids are anteiso-C_15:0_, C_16:1_*ω*7*c*/C_16:1_*ω*6*c*, and iso-C_16:0_. However, after cultivation at 10°C, the proportion of monounsaturated C_16:1_*ω*7*c*/C_16:1_*ω*6*c* fatty acid among the total fatty acids rose to more than 50%. The major respiratory quinones are Q-12 and Q-13.

The type strain is TUM19329^T^ (= GTC 22699^T^ = NCTC 14581^T^), isolated from the freshwater lake sediment of Lake Naga-ike, Skarvsnes, East Antarctica.

## MATERIALS AND METHODS

### Phylogenetic evolutionary analysis and habitat prediction.

The full-length 16S rRNA gene sequences of strain TUM19329^T^ were retrieved from our previous study (8) and then used in a BLASTN search against the NCBI nucleotide collection (nr/nt) database as of July 2021. To further confirm the phylogenetic placement, we also constructed a maximum-likelihood (ML) phylogenetic tree based on multiple alignments of the 16S rRNA gene sequences obtained using MAFFT v7.450 with the auto option, which selected the L-INS-i algorithm (35) and estimation under the GTRGAMMA model using RAxML v8.2.12 software with 100 bootstrap replicates (36). For the genome-based phylogenetic analysis, the genome sequence data of strain TUM19329^T^ and its related species were analyzed using the Type (Strain) Genome Server (TYGS) (accessed July 2021) (37) with the following default settings: all pairwise comparisons among the set of genomes were conducted using the Genome BLAST Distance Phylogeny approach (GBDP), and accurate intergenomic distances were inferred under the algorithm “trimming” and distance formula *d_5_* (38); 100 distance replicates were calculated for each genome; digital DNA-DNA hybridization (DDH) values and confidence intervals were calculated using the recommended settings of GGDC 2 (38); the resulting intergenomic distances were used to infer a balanced minimum-evolution tree with branch support via FASTME v2.1.6.1, including subtree pruning and regrafting (SPR) postprocessing (39); branch support was inferred from 100 pseudobootstrap replicates; the trees were rooted at the midpoint (40) and visualized with PhyD3 (41).

The habitability and distribution of strain TUM19329^T^ and its close relatives were investigated and predicted using the IMNGS platform (12), which conducts a database search against metagenome-derived 16S rRNA gene amplicon data sets, and the ProkAtlas tool, which contains multiple 16S rRNA gene sequences labeled by one environmental category (14). Both tools were performed with a sequence similarity threshold of 99%, using the sequence from strain TUM19329^T^ as the query.

### Morphological, physiological, and biochemical characterization.

For morphological and physiological characterization, TUM19329^T^ was grown on BCYEα agar for 10 days at 25°C. Cell morphology was examined by light microscopy after Gram staining using the Favor G kit (Nissui Pharmaceutical Co., Ltd., Tokyo, Japan). Growth at a range of temperatures (10°C, 15°C, 20°C, 25°C, and 30°C) was assessed in 4 ml of BYE broth supplemented with α-ketoglutarate, l-cysteine, and iron (III) nitrate under constant shaking by transferring a single colony from BCYEα. Growth at various pH values (6.0 to 7.5) and NaCl concentrations (0% to 1.5% [wt/vol]) was also determined with BYE broth. The pH was adjusted by adding either 6 M HCl or 5 M KOH prior to sterilization. The physiological and biochemical characteristics and enzyme activities were tested at 25°C using API 20E and API Campy (bioMérieux, Marcy l’Etoile, France) according to the manufacturer’s instructions, with the exception that the incubation period was extended up to 14 days. The presence of β-lactamase was assessed using Cefinase discs (Becton, Dickinson Microbiology Systems, Sparks, MD, USA).

### Infectivity and intracellular growth assay.

An *Acanthamoeba* sp. previously isolated from a cooling tower in Japan was cultured in 25-cm^2^ tissue culture flasks with 10 ml of PYG broth [2% proteose peptone no. 3, 0.1% yeast extract, 0.1 M glucose, 4 mM MgSO_4_, 0.4 M CaCl_2_, 0.1% sodium citrate, 0.05 M Fe(NH_4_)_2_·6H_2_O, 2.5 mM NaH_2_PO_3_, and 2.5 mM K_2_HPO_3_; pH 6.5] at 25°C for 10 days. Cultures of the *Acanthamoeba* strain were transferred to a 15-ml polypropylene tube, centrifuged at 600 × *g* for 10 min, washed twice with fresh PYG broth, and then adjusted to a titer of 2 × 10^5^ cells ml^−1^. Then, 500 μl of cell suspension was pipetted into each well of a 24-well tissue culture plate (Becton, Dickinson Labware, Franklin Lakes, NJ, USA). After 3 h of incubation at 25°C, the medium was removed and washed three times with Acanthamoeba buffer (AC buffer; PYG broth without proteose peptone, glucose, and yeast extract).

The bacterial suspension was prepared as follows: a single colony of strain TUM19329^T^ that had been incubated on BCYEα agar for 10 days at 25°C was transferred and incubated with continuous shaking in 4 ml of BYE broth supplemented with α-ketoglutarate, l-cysteine, and iron for 5 days until it reached the postexponential phase. L. pneumophila Philadelphia-1 was prepared using the same agar and broth as for TUM19329^T^ but was incubated on the agar for 3 days, followed by shaking in 4 ml of broth for 1 day at 35°C. The bacterial suspension was centrifuged at 3,000 rpm for 10 min and replaced with AC buffer.

Cultures of the *Acanthamoeba* sp. were infected by strain TUM19329^T^ or Philadelphia-1 at a multiplicity of infection (MOI) of 10 and incubated for 1 h after attachment by centrifugation at 500 × *g* for 5 min. At the end of the infection period, nonphagocytosed and nonadherent bacteria were removed by washing three times with fresh AC buffer. The infected *Acanthamoeba* cells were incubated at 25°C and harvested from the bottom of the wells after 0, 24, or 72 h. Then, 100 μl of each sample was fixed onto a microscope slide by cytospin centrifugation at 550 rpm for 1 min and methanol treatment. The slides were stained using Giménez stain (42) and observed by light microscopy. The remaining cell suspensions were sonicated, and then the number of bacteria in each well was calculated as described previously (43).

### Chemotaxonomic characterization.

The chemotaxonomic properties of strain TUM19329^T^ were characterized according to data on the major respiratory quinones, the cellular fatty acid composition, and the genomic GC content. Quinone extraction and determination were performed following a previous method (44). Briefly, cells were grown on BCYEα agar at 25°C for 14 days and were then harvested and freeze-dried. Total lipids were then extracted from the cells using a modified method (45), and the quinones in the crude extract were purified using Sep-Pak plus silica (Waters). The molecular type and concentration of each quinone extracted were analyzed using an ultraperformance liquid chromatography (UPLC) system (Acquity UPLC H-class system, Waters), a photodiode array (PDA) eλ detector (Waters) equipped with an Eclipse plus C_18_ column (2.1 by 150 mm, 1.8 μm; Agilent Technologies), and Masslynx v4.2 software (Waters). The quinone species was finally determined based on the linear relationship between the logarithm of the UPLC retention time and the number of isoprene units according to the equivalent number of isoprene units of quinone components, as reported by Tamaoka et al. (46). The fatty acid composition was measured when cells were cultured at two different temperatures. Cells grown on BCYEα agar at 25°C for 14 days and 10°C for 48 days were harvested and used for analysis. Fatty acid methyl esters were prepared and analyzed using the protocol of the Sherlock Microbial Identification system v6.0 (Microbial ID; MIDI, Inc.). The fatty acid profile was compared and determined using the clinical bacterial library (CLIN6 v6.20). The genomic GC content was calculated from the complete genome sequence of the strain (8).

### Comparative genomic analysis of *Legionella* strains.

Comparative genomic analysis in this study followed the method of a previous study (47). Briefly, the genome sequences of the other 16 *Legionella* spp. were obtained from the EzBioCloud database (48). To eliminate potential differences due to previous analyses using different annotation tools, protein CDSs in all of the genomes were again predicted using Prodigal v2.6.2 (49) of the EzBioCloud whole-genome analysis pipeline with the default settings. The CDSs predicted were classified into clusters of orthologous groups (COGs) based on their functional roles following the reference database eggNOG v4.5 (50). For further functional annotation, they were compared with the Swiss-Prot/UniProt (51) and KEGG (52) databases using UBLAST (53). Using the EzBioCloud comparative genomics pipeline with the default settings, pan-genome orthologous groups (POGs) were determined by a combined reciprocal best-hit method using UBLAST (54) with an E value threshold of 1 × 10^−6^ and an open reading frame-independent method using nucleotide sequences (55) with at least 70% sequence coverage cutoff. After initial grouping, partial short gene sequences were targeted and used for clustering analysis against the determined POGs using UCLUST (53) with a cutoff of ≥95% sequence identity. On the basis of comparison of the CDSs identified in each species and the POGs, the core (common to all), shared (common to two or more species), and unique genes were calculated.

### Codon and amino acid usage.

The codon usage and amino acid usage of the 17 strains were calculated using the Codon Usage Generator v2.4 (http://bioinfo.ie.niigata-u.ac.jp/?Codon%20Usage%20Generator) (56, 57). The created data sets were analyzed and visualized using Python v3.7.1 (https://www.python.org) and its libraries, such as pandas v1.1.1, matplotlib v3.3.1, and seaborn v0.10.1.

### Detection of ISs.

Genes in COG category [L] classified into four of the COGIDs (COG2801, COG2963, COG1484, and COG4584), which were especially enriched in TUM19329^T^, were phylogenetically analyzed to clarify the sequence relationship. The nucleotide sequences of genes classified in each COG ID were aligned using MAFFT v7.450 using the auto option, which selected the L-INS-i algorithm (35). Subsequently, maximum-likelihood (ML) phylogenetic trees with 100-bootstrap replicates were estimated according to the GTRGAMMA model using RAxML software v8.2.12 (36) and were visualized using FigTree v1.4 (http://tree.bio.ed.ac.uk/software/figtree/). Phylogenetic analysis showed that many of the gene sequences classified into each COGID were duplicated sequences. Duplicated gene sequences that formed clusters in the phylogenetic tree always had another gene that was detected in pairs in the genome. Using the locus information of these paired gene sequences, the paired regions and their upstream and downstream regions (500 bases each) were extracted using a Python script. The extracted sequences were aligned using MAFFT with the auto option, which selected the L-INS-i algorithm (35), and then manually trimmed to identify the full length of the ISs. The structure of each IS and its location in the genome were visualized using the Python module, GenomeDiagram (58), and Biopython v1.74 (59). The full-length ISs are available on Figshare (see “Data Availability”).

### Detection of horizontal gene transfer.

HGT events were identified using HGTector, a sequence similarity-based HGT prediction pipeline (33). A protein sequence similarity search was performed using DIAMOND v2.0.4 (60) against a database (generated by HGTector) that contains one representative per species from all available nonredundant RefSeq prokaryotic proteomes (October 2019). *Legionella* was set as the self group, and *Legionellales* was set as the close group. Quality cutoffs for valid hits were an E value of ≤1e-10, percentage identity of ≥30%, and query coverage of ≥70%. For each protein-coding gene, the top 100 highest-scoring hits from different species were retained.

### Data availability.

The genome sequence of *Legionella antarctica* TUM19329^T^ was previously deposited in DDBJ/ENA/GenBank under the accession number AP022839, and the raw sequence data for this sequence were deposited under SRA accession numbers DRR213975 (MiSeq) and DRR213976 (MinION) (8). The data and script for the analysis presented in this article are available on Figshare (doi: 10.6084/m9.figshare.15912081).
