# Supplementary material for: Characterization of the First Cultured Psychrotolerant Representative of Legionella from Antarctica Reveals Its Unique Genome Structure
Source: Microbiol Spectr. 2021 Oct 20;9(2):e00424-21. doi: 10.1128/Spectrum.00424-21 (PMC8528123; doi:10.1128/Spectrum.00424-21)
Supplement: SUPPLEMENTAL FILE 1 — Supplemental material. Download SPECTRUM00424-21_Supp_1_seq6.pdf, PDF file, 0.3 MB [file spectrum00424-21_supp_1_seq6.pdf]

Supplementary Information

Table S1. Number of protein coding genes classified into each functional COG category. Each alphabet represents functional categories as same as those shown in Fig. 4.

Table S2. List of Mobile gene elements found in TUM19329^T

Table S3. List of Insertion sequences (ISs) structures interrupted by other ISs

Supplementary Figure legends

Fig. S1. Maximum-Likelihood tree of the TUM19329^T with related *Legionella* spp. based on 16S rRNA genes. Bootstrap value based on 100 resamples are shown. Scale bar indicates the base substitutions for each position. *Coxiella burnettii* (Accession No. HM208383) was used as an out group.

Fig. S2. Growth curves of strain TUM19329^T in BYE broth at different (a) NaCl concentration and (b) pH.

Fig. S3. Maximum-likelihood tree of the TUM19329^T with related *Legionella* spp. based on functional homologs of IcmRs. Bootstrap value based on 100 resamples are shown. Scale bar indicates the base substitutions for each position.

Fig. S4. Example of insertion sequence interrupting other gene.

ISLa5 interrupting ISLa1 at position 91894 to 95878 of the chromosome of TUM19329^T.

Table S1

Taxon name	CELLULAR PROCESSES AND SIGNALING								INFORMATION STORAGE AND PROCESSING					METABOLISM								POORLY CHARACTERIZED
	D	M	N	O	T	U	V	Z	A	B	J	K	L	C	E	F	G	H	I	P	Q	S
TUM19329^{T}	29	150	35	88	69	49	34	0	1	1	148	67	**721**	115	133	42	68	73	91	83	34	802
Legionella bozemanae	30	177	34	99	108	89	45	1	1	1	159	111	206	133	187	56	97	81	98	128	61	893
Legionella cincinnatiensis	26	163	1	112	87	52	50	1	1	0	156	107	193	132	168	57	88	79	87	109	60	892
Legionella fallonii	29	197	50	112	102	61	64	0	1	2	162	107	171	152	193	56	89	90	95	104	53	972
Legionella hackeliae	27	163	35	97	77	50	50	1	1	1	159	99	143	138	165	47	86	78	72	80	44	797
Legionella jordanis	27	152	36	100	69	54	29	1	1	1	152	68	149	124	146	49	84	69	70	88	37	715
Legionella lansingensis	26	147	1	99	51	44	35	1	1	2	147	64	156	126	168	51	82	77	70	79	40	642
Legionella longbeachae	26	178	1	117	106	55	50	0	1	0	150	103	189	155	178	59	86	80	85	112	53	861
Legionella micdadei	24	163	50	105	89	46	30	1	1	0	150	78	122	138	157	54	77	77	82	83	44	685
Legionella moravica	28	153	50	104	93	57	42	2	1	0	150	88	158	129	152	58	73	75	90	98	36	804
Legionella pneumophila	26	151	34	109	82	56	39	0	1	0	152	95	145	137	165	55	80	74	91	96	41	769
Legionella quateirensis	30	170	52	110	99	89	37	3	1	1	155	109	160	141	182	60	89	78	95	102	56	909
Legionella sainthelensi	26	179	1	122	109	53	51	0	1	1	158	108	220	148	188	57	95	80	88	127	63	908
Legionella shakespearei	28	136	36	94	72	56	38	0	1	0	148	86	130	123	169	55	72	70	84	78	51	695
Legionella tucsonensis	27	154	34	100	79	40	29	0	1	0	152	77	138	134	164	55	85	79	86	96	44	694
Legionella wadsworthii	27	166	2	110	96	47	41	0	1	1	152	92	120	132	174	57	105	84	86	103	42	764
Legionella worsleiensis	27	134	52	97	71	50	29	1	1	1	150	78	155	117	123	46	67	70	69	70	36	638

Table S2

COG IDs	Description	Number of genes
L:COG2801	Retrotransposon protein	163
L:COG2963	Transposase	156
L:COG4584	Transposase	45
L:COG1484	IstB domain protein ATP-binding protein	41
L:COG3436	Transposase	31
L:COG3344	RNA-directed DNA polymerase	23
L:COG0582	Integrase	15
L:ENOG4110SJK	Transposase	15
L:COG4644	Transposase	12
L:ENOG4111NDY	Transposase	11
L:ENOG4111UQ5	Transposase	11
L:COG3316	Transposase	11
L:COG5433	Transposase	10
L:COG3464	Transposase	9
L:COG1961	Resolvase	6
L:COG3293	Transposase	5
L:COG3039	Transposase	5
L:COG3335	Transposase	5
L:ENOG410XS43	Transposase	4
L:ENOG4111H7A	Transposase	4
L:ENOG410ZVYF	Transposase IS4 Family Protein	4
L:ENOG41103Q3	Integrase	4
L:ENOG4111KXU	Transposase (IS4 family)	3
L:ENOG410XPNN	Transposase	2
L:ENOG410XT8A	Transposase	2
L:ENOG410XP6T	integrase catalytic	1
L:ENOG410XPK3	Integrase catalytic subunit	1
L:ENOG410XRAH	Transposase	1
L:ENOG4111IPS	Transposase	1
L:ENOG4111ZAW	Transposase	1
L:COG3385	Transposase	1
L:COG3547	Transposase	1
L:COG3666	Transposase	1
L:COG2826	Transposase	1
L:COG3328	Transposase	1
L:ENOG410XQBA	Transposase	1
L:ENOG4111USC	Transposase (IS4 family)	1
L:ENOG411218M	Transposase	1
L:ENOG41123GT	IS4 family Transposase	1

Table S3

Fragmented IS elements	Description
TUM19329_00105	Fragments of ISLa1 interrupted by ISLa5
TUM19329_00310	Fragments of ISLa1 interrupted by ISLa1
TUM19329_01099	Fragments of ISLa1 interrupted by ISLa1
TUM19329_01101	Fragments of ISLa1 interrupted by ISLa4
TUM19329_01105	Fragments of ISLa1 possibly interrupted by other mobile elements
TUM19329_01438	Fragments of ISLa1 interrupted by ISLa1
TUM19329_01875	Fragments of ISLa1 interrupted by ISLa1
TUM19329_01878	Fragments of ISLa1 interrupted by ISLa1
TUM19329_02272	Fragments of ISLa1 interrupted by ISLa1
TUM19329_02541	Fragments of ISLa1 possibly interrupted by other mobile elements
TUM19329_02862	Fragments of ISLa1 possibly interrupted by other mobile elements
TUM19329_00070, TUM19329_00071	Fragments of ISLa5 possibly interrupted by other mobile elements
TUM19329_01569, TUM19329_01570	Fragments of ISLa5 possibly interrupted by other mobile elements
TUM19329_02486	Fragments of ISLa5 interrupted by ISLa1
TUM19329_02722	Fragments of ISLa5 interrupted by ISLa1
TUM19329_03355	Fragments of ISLa5 interrupted by ISLa1
TUM19329_00827	Fragments of ISLa2 interrupted by ISLa1
TUM19329_01256	Fragments of ISLa2 interrupted by ISLa4
TUM19329_01982	Fragments of ISLa2 interrupted by ISLa4
TUM19329_01985	Fragments of ISLa2 interrupted by ISLa4
TUM19329_01992	Fragments of ISLa2 possibly interrupted by other mobile elements
TUM19329_02265	Fragments of ISLa2 interrupted by ISLa4
TUM19329_02268	Fragments of ISLa2 interrupted by ISLa4
TUM19329_02417, TUM19329_02420	Fragments of ISLa2 interrupted by ISLa4
TUM19329_00100	Fragments of ISLa3 interrupted by ISLa2
TUM19329_02845	Fragments of ISLa3 possibly interrupted by other mobile elements
TUM19329_02979	Fragments of ISLa3 interrupted by ISLa3
TUM19329_03005	Fragments of ISLa3 interrupted by ISLa3
TUM19329_03006	Fragments of ISLa3 interrupted by ISLa3
TUM19329_00108	Fragments of ISLa1 interrupted by ISLa5
TUM19329_02275	Fragments of ISLa1 interrupted by ISLa1
TUM19329_02537	Fragments of ISLa1 possibly interrupted by other mobile elements
TUM19329_02900	Fragments of ISLa3 interrupted by ISLa3
TUM19329_02490	Fragments of ISLa5 possibly interrupted by other mobile elements
TUM19329_03082	Fragments of ISLa5 possibly interrupted by other mobile elements
TUM19329_03352	Fragments of ISLa5 interrupted by ISLa1

Fig. S1

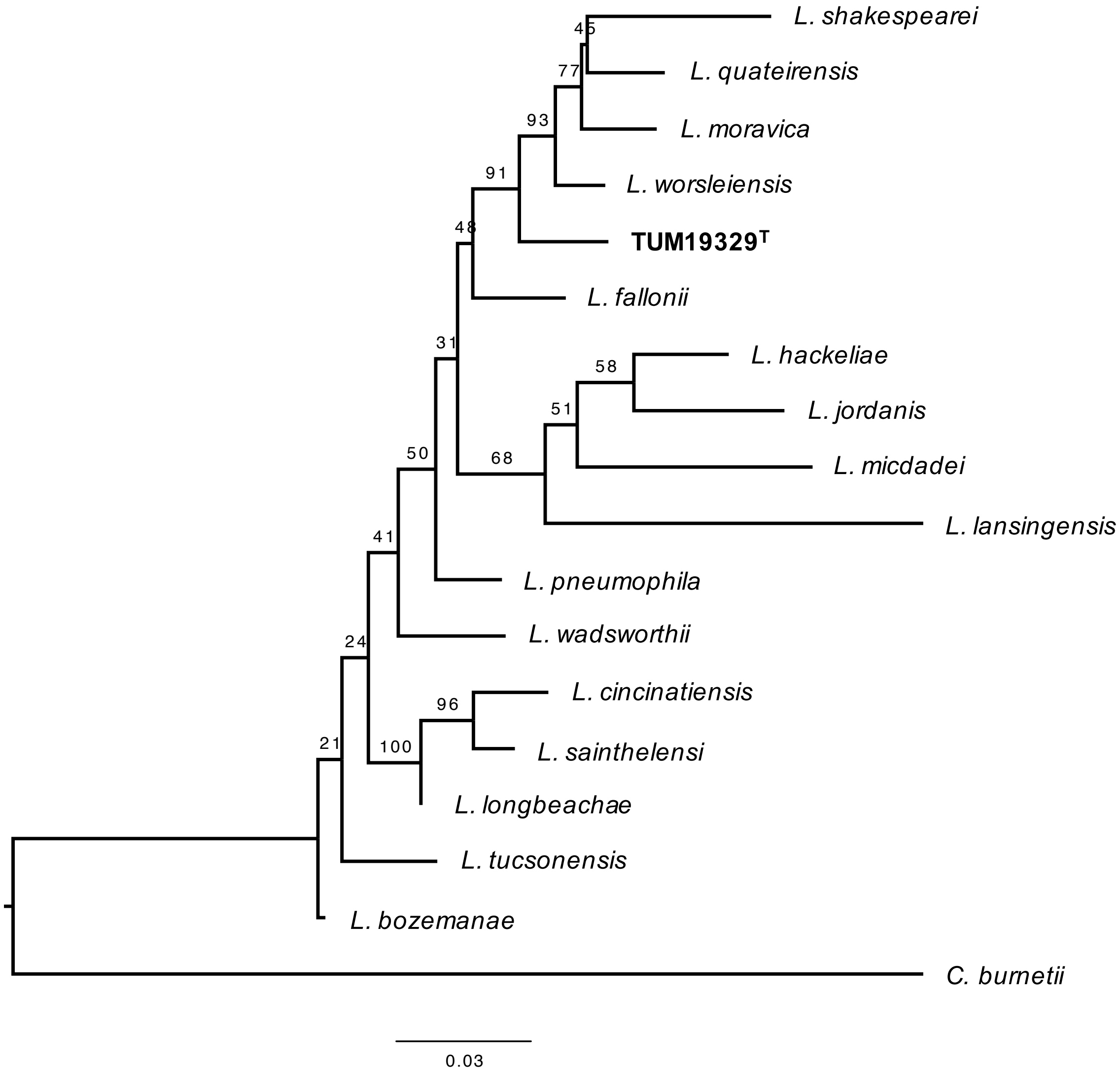
L. shakespearei
45
L. quateirensis
77
L. moravica
93
91
L. worsleiensis
48
TUM19329T
L. fallonii
31
L. hackeliae
58
L. jordanis
51
50
68
L. micdadei
L. lansingensis
41
L. pneumophila
L. wadsworthii
24
L. cincinatiensis
96
21
100
L. sainthelensi
L. longbeachae
L. tucsonensis
L. bozemanae
C. burnetii
0.03

Fig. S2

(a)

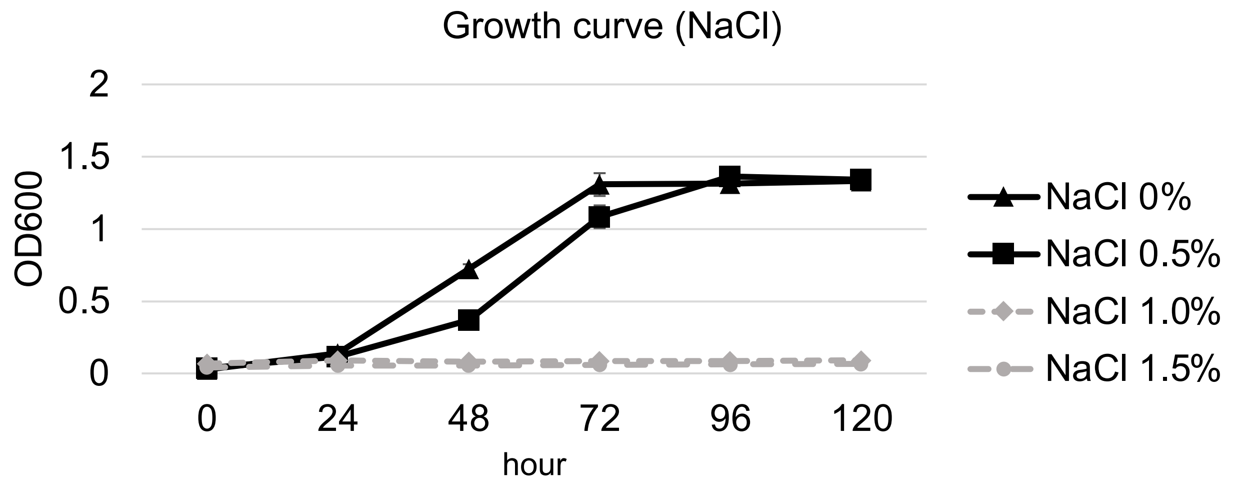

Growth curve (NaCl)
OD600
2
1.5
1
0.5
0
0
24
48
72
96
120
hour
NaCl 0%
NaCl 0.5%
NaCl 1.0%
NaCl 1.5%

(b)

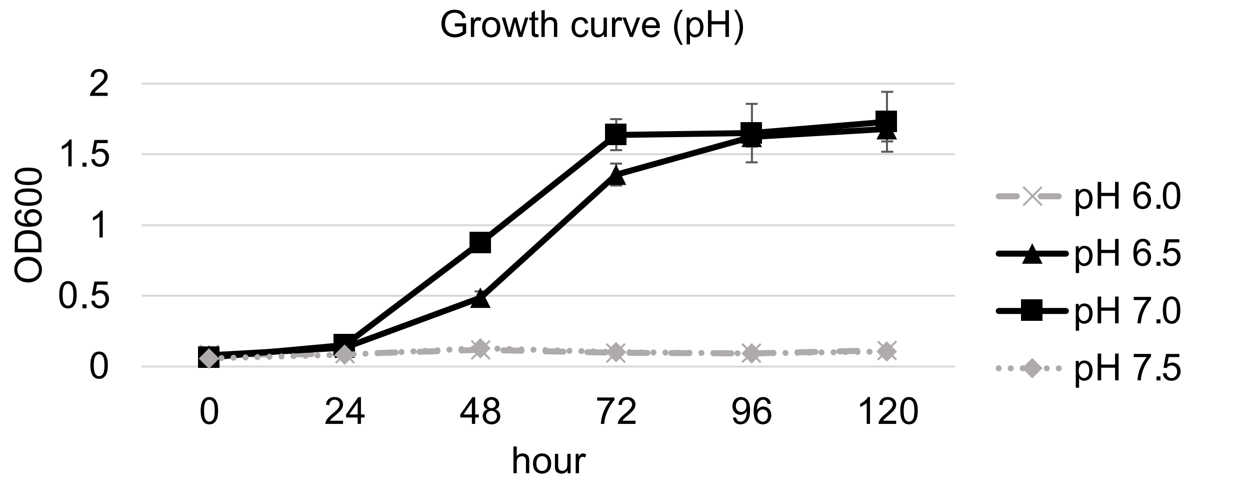

Growth curve (pH)
OD600
2
1.5
1
0.5
0
0
24
48
72
96
120
hour
pH 6.0
pH 6.5
pH 7.0
pH 7.5

Fig. S3

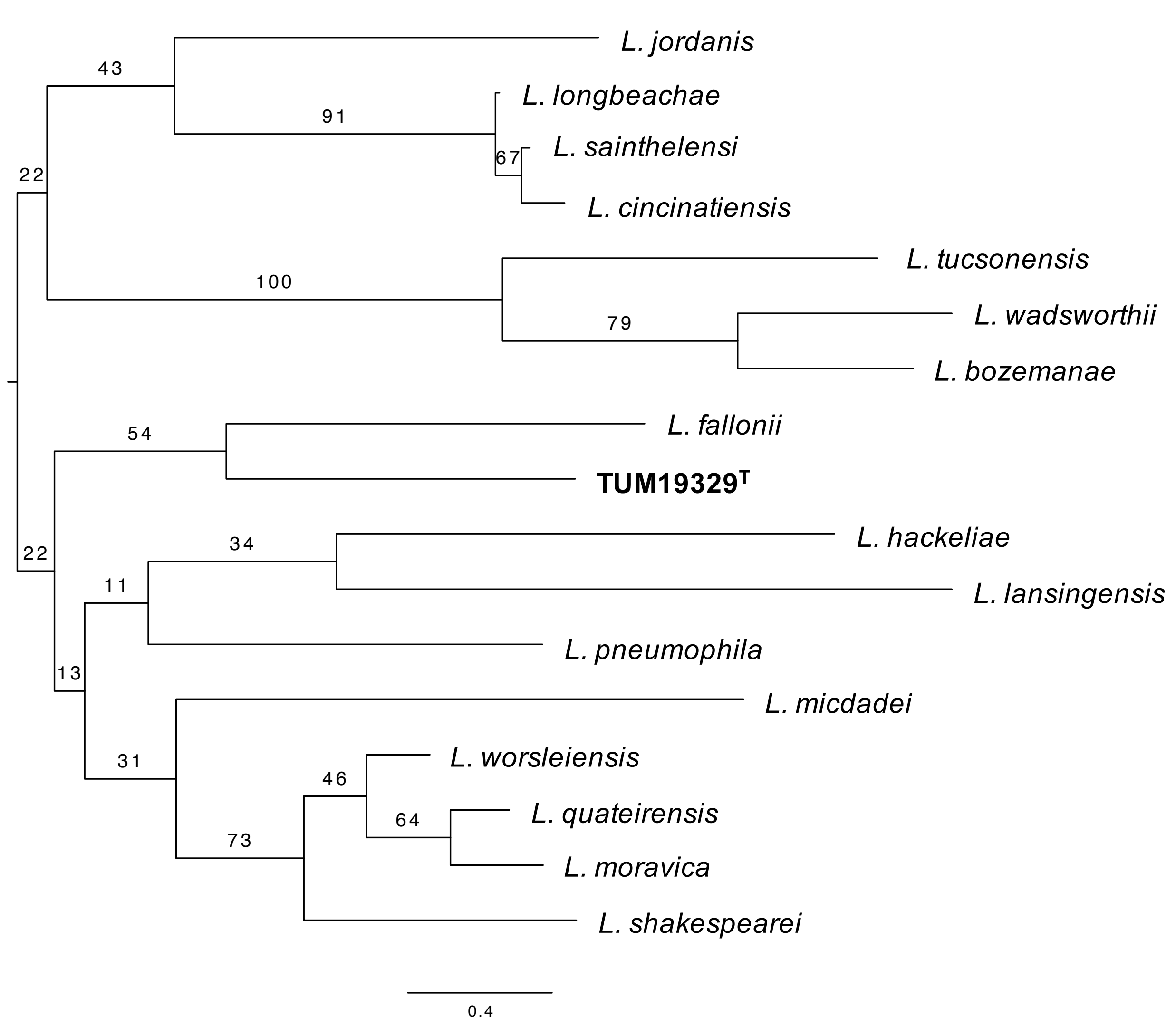
L. jordanis
L. longbeachae
L. sainthelensi
L. cincinatiensis
L. tucsonensis
L. wadsworthii
L. bozemanae
L. fallonii
TUM19329T
L. hackeliae
L. lansingensis
L. pneumophila
L. micdadei
L. worsleiensis
L. quateirensis
L. moravica
L. shakespearei
43
91
67
22
100
79
54
22
34
11
13
31
46
64
73
0.4

Fig. S4

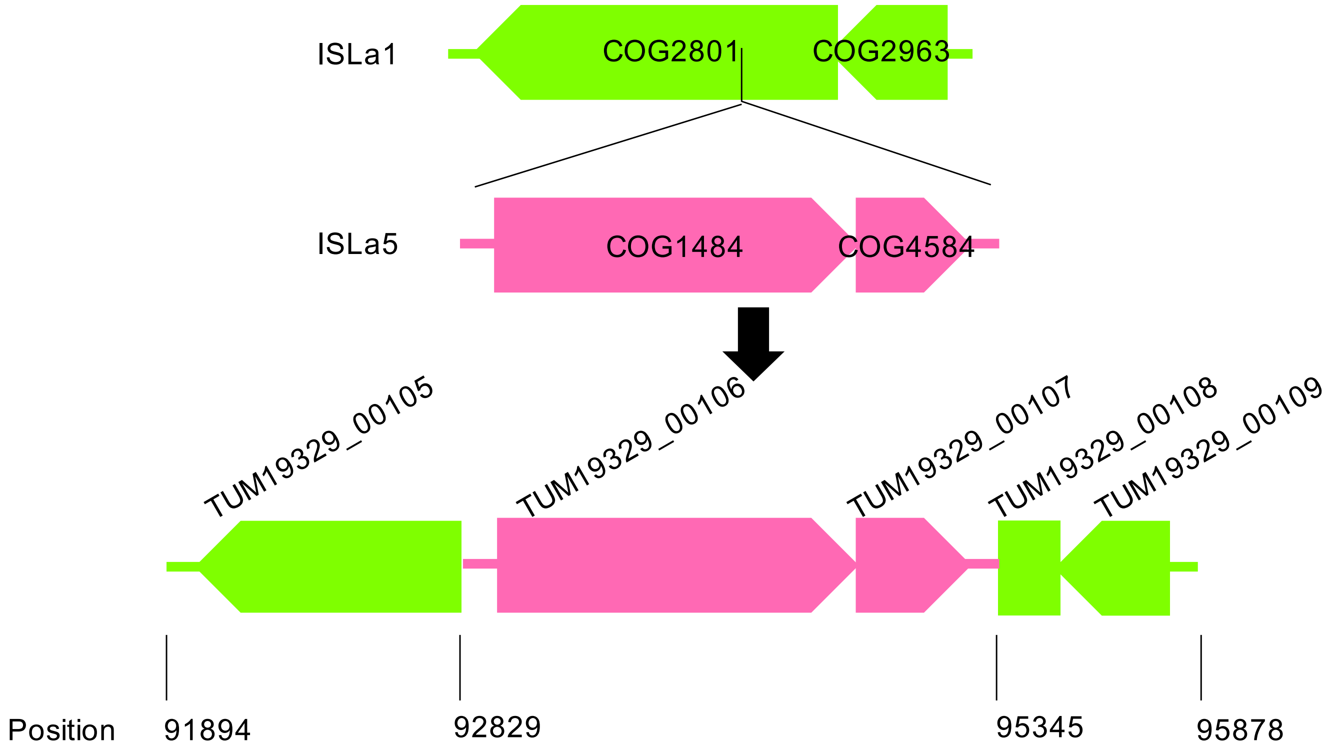
ISLa1
COG2801
COG2963
ISLa5
COG1484
COG4584
TUM19329_00105
TUM19329_00106
TUM19329_00107
TUM19329_00108
TUM19329_00109
Position
91894
92829
95345
95878
